# What makes online teaching spatial? Examining the connections between K-12 teachers’ spatial skills, affect, and their use of spatial pedagogy during remote instruction

**DOI:** 10.1186/s41235-022-00377-7

**Published:** 2022-03-21

**Authors:** Kelsey Rocha, Catherine M. Lussier, Kinnari Atit

**Affiliations:** School of Education, University of California, Riverside, 1207 Sproul Hall, Riverside, CA 92521 USA

**Keywords:** Teacher cognition, Spatial skills, Spatial anxiety, Online pedagogy, K-12 education

## Abstract

Spatial skills are critical for student success in K-12 STEM education. Teachers’ spatial skills and feelings about completing spatial tasks influence students’ spatial and STEM learning at both the primary and secondary levels. However, whether spatial skills and spatial anxiety differ or not between these two teacher levels is unknown. Additionally, the relations between teachers’ spatial skills, spatial anxiety, and their use of spatial pedagogical practices in remote learning settings is unknown. Here, we investigated if spatial skills and spatial anxiety differ between teachers working at primary versus secondary levels, and examined the relations between their spatial skills and spatial anxiety while accounting for additional influential factors—general reasoning ability and general anxiety. Lastly, we investigated how teachers’ spatial skills in conjunction with their spatial anxiety relate to their use of spatial teaching practices for online instruction. Sixty-two K-12 teachers completed measures of spatial skills, spatial anxiety, general anxiety, general reasoning, and a teaching activities questionnaire. Results indicate that spatial skills and spatial anxiety may not vary between teachers working at primary versus secondary levels, but that higher spatial skills in teachers are associated with lower spatial anxiety for mental manipulation tasks. Additionally, teachers with weaker spatial skills and lower mental manipulation anxiety reported more frequently using spatial teaching practices when teaching remotely due to COVID-19. These findings may have broad implications for teacher professional development with regards to developing students’ spatial skills during remote learning.

## Statement of significance

Spatial skills—the set cognitive skills used when reasoning about objects in real and imagined spaces—have been shown to be a significant contributor to STEM learning and success across the K-12 educational level. Further, it has been established that spatial skills are malleable, meaning that efforts to bolster this set of skills may result in their improvement. Thus far, many efforts aimed at improving students’ spatial skills have been through direct, student-centered interventions. This method fails to take into account teachers and the impact that they may have on their students’ acquisition of these skills. There is copious evidence that suggests that teachers’ cognitive abilities, affect, and attitudes play an important role in their students’ learning outcomes and experiences. The study presented here examines the relations between K-12 teachers’ spatial skills, their level of anxiety when engaging in spatial tasks, and their use of spatial teaching practices while conducting remote instruction during COVID-19. Understanding the relations between these factors is imperative for bridging the evident gap between researcher-crafted interventions aimed at improving students’ spatial skills and teachers’ use of spatial pedagogical practices in their teaching.

## Introduction

The need for career ready college students in science, technology, engineering, and mathematics (STEM) disciplines is projected to continue to exponentially grow and so is of ongoing national interest (Executive Office of the President, 2018; US Department of Education, 2013). However, kindergarten to 12th grade (i.e., K-12) students are often underprepared to pursue STEM subjects further as they transition from K-12 into higher education (National Center for Education Statistics, 2019). Many of the efforts aimed at improving K-12 students’ performance in STEM disciplines have focused on identifying the factors contributing to students’ learning and outcomes in the various relevant domains (e.g., Burte et al., 2017; Frick, 2019). As a result of these efforts, spatial skills have been identified as a cognitive skill set critical to students’ learning and success in STEM disciplines (e.g., Hodgkiss et al., 2018; Wai et al., 2009). Spatial skills allow us to navigate and manipulate objects in real and imaginary spaces and are central to understanding and reasoning about STEM concepts taught across the K-12 educational levels (e.g., Gilligan et al., 2017; Hodgkiss et al., 2018). For example, spatial skills allow us to visualize basic concepts such as that two parts of a cookie can be brought together to make a whole cookie, as well as more complex concepts such as how the earth rotates around the sun while also rotating around its axis. Moreover, spatial skills are malleable and can be improved with training and practice (e.g., Uttal et al., 2013). There is much evidence to support that teachers play a critical role beyond that of delivering content in K-12 students’ educational experiences and outcomes (e.g., Ball et al., 2005; den Brok et al., 2004; Perera & John, 2020), and that teachers’ skills and feelings influence their pedagogy (Fennema et al., 1990; Otumfuor & Carr, 2017). However, only a few studies have examined how teachers’ spatial skills and comfort with completing spatial tasks (i.e., spatial anxiety) influence their use of spatial practices in their teaching (e.g., Atit & Rocha, 2020; Gagnier et al., 2021; Gunderson et al., 2013) and none have looked at how these factors influence teachers’ use of spatial practices in online teaching, specifically. As classroom experiences–physical and virtual ones–are potentially a critical source of students’ spatial learning at the K-12 level, here we aim to gain a better understanding of the relations between teachers’ spatial skills, spatial anxiety, and spatial pedagogical practices while conducting remote instruction during the COVID-19 pandemic.

## Why is K-12 spatial learning important?

Spatial skills are fundamentally linked to students’ STEM outcomes at all levels of K-12 education (Cheng & Mix, 2014; Wai et al., 2009). Specifically, this class of skills has been found to predict mathematics and science understanding at both the primary (e.g., Geer et al., 2019; Gunderson et al., 2013; Hodgkiss et al., 2018) and secondary levels (Ganley et al., 2014; Stavridou & Kakana, 2008). Spatial skills also predict students’ pursuit of STEM degrees and occupations later in life. Spatial skills measured in middle school (Shea et al., 2001) and high school (Wai et al., 2009) predict STEM readiness and performance anxiety levels (Lauer et al., 2018; Sokolowski et al., 2019), and STEM degree attainment and STEM employment more than a decade later (see Garcia et al., 2021 for a systematic review). Even at the expert level, STEM professionals often employ spatial skills when completing tasks within their domain (see Atit et al., 2020 for a more complete review). For example, petroleum geologists use spatial skills when deciding on the location for a new oil well. They interpret and visualize the shapes and locations of three-dimensional (3D) geologic structures that exist under the ground from two-dimensional (2D) seismic data.

A meta-analysis synthesizing the findings of 217 studies examining the effect of training on participants’ spatial skills revealed that spatial skills can be improved through practice. Moreover, results showed that the effects of training are not limited to the task at hand and are long lasting. The learning gains acquired through training on a particular kind of spatial task (e.g., mental rotation—an intrinsic dynamic spatial task) were found to transfer to other similar spatial tasks (e.g., paper folding—another intrinsic dynamic spatial task), as well as those that fall within a distinct spatial category (e.g., water level task—an extrinsic static spatial task; Uttal et al., 2013).

In addition to systematic and deliberate training, evidence from correlational studies suggests that experiences engaging in spatial tasks are related to participants’ performance on psychometric measures of spatial skills. Frequency of informal spatial play with puzzles, blocks, and board games in four- to seven-year-old children was related to performance on the Block Design subtest of the Wechsler Preschool and Primary Scale of Intelligence—Fourth Edition, while other types of play and parent–child activities were not related to Block Design score (Jirout & Newcombe, 2015). In another study, the frequency and quality of puzzle play in children ages 26–46 months predicted their performance on a mental transformation test at 54 months (Levine et al., 2012).

Furthermore, some research conducted in the last decade has found that practicing the completion of spatial tasks results in improvement in students’ STEM outcomes (Cheng & Mix, 2014; Hawes et al., 2022; Miller & Halpern, 2013). Cheng and Mix (2014) found that practice in solving mental rotation problems leads to better addition and subtraction problem solving skills in primary students. Similarly, spatial training in undergraduates has been found to improve their performance on introductory STEM coursework (Miller & Halpern, 2013; Sorby et al., 2018). However, the findings on determining if practicing spatial skills bolsters students’ STEM performance are mixed. Some studies have failed to find causal relations between spatial training and students’ STEM outcomes (e.g., Cornu et al., 2019; Hawes et al., 2015), indicating that the details of how, why, and when spatial training can bolster students’ STEM learning and performance are not well understood.

In sum, spatial skills are fundamental to STEM learning at all educational levels (e.g., Ganley & Vasilyeva, 2011; Gunderson et al., 2012; Miller & Halpern, 2013), and spatial skills in grade school predict STEM outcomes in adulthood (e.g., Garcia et al., 2021; Shea et al., 2001; Wai et al., 2009). Moreover, spatial skills are malleable and can be improved through practice and experiences engaging in spatial tasks (e.g., Uttal et al., 2013). Thus, the development of strong spatial skills early in students’ educational trajectories may have long term impacts on their future STEM success and retention.

## The relations between teachers’ skills, affect, and pedagogy

While curriculum standards may be similar across grade levels [e.g., Next Generation Science Standards (NGSS Lead States, 2013); Common Core State Standards (National Governors Association Center for Best Practices and Council of Chief State School Officers, 2010], the methods and strategies teachers choose to convey the specified content can vary greatly. For instance, the concept of mathematical equivalence can be taught to primary students visually, such as by using a balance to demonstrate that the two sides of the equation need to be the same, or it can be taught numerically by emphasizing that the numerical result of the equation on each side of the equal sign needs to be the same. Evidence suggests that teachers’ domain knowledge and feelings about the domain, in conjunction with their pedagogical content knowledge (Shulman, 1986), influence their pedagogical practices (e.g., Fennema et al., 1990; Nespor, 1987), and can be identified as early as the pre-service teacher stage (Novak & Tassell, 2017). Teachers with low understanding for a topic employ more didactic practices, relying on the textbook and encouraging students to learn the material within it. Similarly, teachers with a low self-concept in the domain also report using more didactic approaches to teaching, and express that they lack the time, interest, or motivation to try new teaching strategies (e.g., Relich, 1996). On the other hand, teachers with high understanding for a topic and/or high self-concepts in the domain encourage questions from the students and promote active and student-centered learning (e.g., Grossman, 1990; Harlen, 1997; Osborne & Simon, 1996).

One limitation of this research is that much of the focus on understanding the relations between teachers’ skills, affect, and pedagogical practices has been within the domains which are part of the formal K-12 curriculum, such as mathematics (e.g., Beilock et al., 2010; Burte et al., 2020; Hill, et al., 2005), science (e.g., Novak & Wisdom, 2018), and literacy (Grossman, 1990). Only a handful of studies have considered the teacher’s role in informal domains that are relevant to pedagogy across the K-12 curriculum, specifically spatial learning (e.g., Atit & Rocha, 2020; Gagnier et al., 2021; Gunderson et al., 2013; Otumfuor & Carr, 2017). This is problematic because recent research indicates that many teachers feel unprepared to conduct spatial learning in their classroom (Power & Sorby, 2020), meaning that any efforts at bolstering K-12 students’ spatial skills through classroom practices and experiences (e.g., Lauer et al., 2017; Sorby, 2009) may only have limited effects. Gaining a better understanding of how teachers’ spatial skills and their comfort in completing spatial tasks relate to their use of spatial practices in their teaching potentially has broad implications for the development of K-12 students’ spatial skills.

## What we already know about teachers’ spatial skills, spatial anxiety, and their teaching

Even though spatial learning is not conventionally a formal part of the K-12 curriculum, evidence suggests that teachers have influence on the development of their students’ spatial skills, as well as their engagement in spatial learning experiences (Gunderson et al., 2013; Otumfuor & Carr, 2017). A study by Gunderson et al. (2013) found that teachers’ spatial anxiety significantly predicted their students’ spatial learning. High spatial anxiety in primary teachers resulted in lower student performance on a Mental Rotations Test at the end of the year–even after controlling for students’ spatial skills at the beginning of the year, their phonological working memory, grade level, and teachers’ math anxiety. Otumfuor and Carr (2017) found that teachers’ spatial skills are positively related to their use of spatial pedagogical practices for in-person learning settings. For example, middle school teachers’ spatial skills, in conjunction with their pedagogical content knowledge, were related to the use of graphs and diagrams in their geometry instruction. Furthermore, teachers’ spatial skills were also related to their use of representational gestures in their teaching.

More recently, building on prior work by Gunderson et al. (2013) and Otumfuor and Carr (2017), Atit and Rocha (2020) examined the relations between teachers’ spatial skills, spatial anxiety, and their reported use of spatial practices, such as using graphs and diagrams, in their in-person teaching. K-12 teachers completed the Mental Manipulation and Imagery subscales of a spatial anxiety measure (Lyons et al., 2018), a mental rotation task, and a teaching activities questionnaire. Results revealed that teachers’ mental rotation skills were negatively associated with their spatial anxiety for completing mental manipulation tasks, and positively associated with their reported use of spatial pedagogical practices (Atit & Rocha, 2020).

While this research provides a promising start to understanding the role of teachers’ spatial skills and anxieties on students’ spatial learning, there is still much left to be understood. Particularly, in the study by Atit and Rocha (2020), no direct measure of teachers’ general reasoning skills was administered. This is problematic because prior research indicates that the relations between spatial skills and spatial anxiety vary depending on participants’ general reasoning abilities. Ramirez et al. (2012) have found that the negative relations between spatial skills and spatial anxiety are stronger for individuals with higher working memory capacity. Working memory is a strong indicator of general reasoning skills (Kyllonen & Christal, 1990; Kyllonen & Dennis, 1996). Similarly, Atit and Rocha (2020) also did not measure teachers’ general anxiety. Participants’ level of general anxiety could account for some of the variance attributed to the relations found between teachers’ spatial skills and spatial anxiety in this prior research (Atit & Rocha, 2020), even though it is not specific to spatial processing (e.g., Sokolowski et al., 2019). Lastly, Atit and Rocha (2020) only examined teachers’ spatial anxiety for completing small-scale spatial tasks and did not examine teachers’ anxiety for completing large-scale spatial tasks. Small-scale spatial tasks involve the visualization and manipulation of 2D and 3D forms derived from polygons, such as mental rotation (Carroll, 1993; Oltman et al., 1971; Shepard & Metzler, 1971). Distinct from small-scale spatial tasks are large-scale spatial tasks, which are context dependent and require solving spatial problems that are situated within the environment (Allen et al., 1996; Weisberg & Newcombe, 2016; Weisberg et al., 2014). Examples of large-scale spatial tasks include finding one’s way in the environment or learning the layout of a building or city (Hegarty & Waller, 2004). As the skills used when completing small-scale spatial tasks are separable from those used when completing large-scale spatial tasks (Hegarty & Waller, 2004), perhaps the level of anxiety teachers’ experience for each type of task also differs. Thus, a more precise understanding of the relations between teachers’ spatial skills and spatial anxiety has yet to be ascertained.

To our knowledge, only one study has ever examined if teachers’ spatial skills differ by the educational level in which they teach. Using a nationally representative dataset, Atit et al. (2018) examined the spatial skills of high school students who later became pre-college teachers. Results showed that secondary STEM teachers had substantially stronger spatial skills than secondary non-STEM teachers and preschool/primary teachers. Compared to the general population, 79% of secondary-STEM teachers had above average spatial skills versus 61% of secondary non-STEM teachers and 47% of preschool/primary teachers. One limitation of this study is that the data examined were collected on students in 1960 (Atit et al., 2018). If differences in spatial skills between different kinds of teachers persist today, it has yet to be examined.

Much research indicates that spatial skills are fundamental for students’ STEM learning at both the primary (e.g., Battista & Clements, 1996; Guay & McDaniel, 1977; Gunderson et al., 2012) and secondary educational levels (e.g., Atit et al., 2020; Ganley et al., 2014). Moreover, content and practices that heavily engage students’ spatial skills are ubiquitous throughout the K-12 STEM curricula. For instance, although spatial skills are not explicitly stated in the NGSS, the mastery of science/engineering content and practices that heavily rely on spatial thinking are included in the standards for each grade-level. For example, kindergarten standards include developing skills in using and developing models (i.e., diagrams, drawings) to represent concrete events or design solutions. High school standards include developing skills to use a model to predict the relationships between systems or between components of a system (National Research Council, 2012). Teachers’ characteristics influence their choice in pedagogical strategies (e.g., Atit & Rocha, 2020; Johnston & Ahtee, 2006; Ozden, 2008) as well as their students’ learning in the domain (e.g., Beilock et al., 2010; Otumfuor & Carr, 2017). Consistent with prior research (Atit et al., 2018), if a disparity between primary and secondary teachers’ spatial skills persists, with primary teachers having weaker spatial skills than their secondary counterparts, this finding may have broad implications for the development of students’ early spatial and STEM learning today. Thus, identifying differences in spatial skills between different kinds of teachers would inform as to which kinds of teachers may benefit from additional pedagogical support specific to spatial learning.

Lastly, no studies have examined the use of teachers’ spatial pedagogical practices in remote instructional settings. Research on teachers’ use of spatial teaching practices has only been conducted in scenarios of in-person instruction. Thus, teachers’ use of spatial practices when teaching in online environments is unknown. The sudden shift to entirely remote instruction due to the COVID-19 pandemic may have exacerbated many teachers’ anxieties and insecurities with integrating technology into their teaching (i.e., computer anxiety; e.g., Henderson & Corry, 2021; Orlando, 2014). Teachers’ computer anxiety coupled with the limitations associated with delivering online instruction (e.g., abbreviated instructional time, students’ limited attention span during online learning) may have influenced their pedagogical choices. Thus, teachers’ use of spatial pedagogical practices in remote settings may vary from their use during in-person settings. With the increased reliance on K-12 remote instruction due to the COVID-19 pandemic (Archambault & Borup, 2020; Boltz et al., 2021), identifying factors contributing to students’ spatial learning in online learning settings could have broad implications for students’ STEM learning outcomes.

## The current study

Building on prior research (Atit & Rocha, 2020; Atit et al., 2018), first we examine if teachers’ spatial skills and spatial anxiety differ for different types of teachers (i.e., primary versus secondary). Informed by Atit et al.’ (2018) findings that preschool/primary teachers have weaker spatial skills than secondary STEM teachers, we hypothesize that in our study primary teachers will exhibit weaker spatial skills than secondary teachers. Second, we examine the relations between K-12 teachers’ spatial skills and their spatial anxiety, while accounting for additional influential factors—general reasoning ability and general anxiety. We hypothesize that in this study we will replicate prior findings showing that teachers with weaker spatial skills have higher spatial anxiety (Atit & Rocha, 2020). Lastly, we investigate how teachers’ spatial skills in conjunction with their spatial anxiety relate to their use of spatial practices during remote teaching. Results from studies examining in-person instruction indicate that teachers with stronger spatial skills make greater use of spatial practices in their teaching (e.g., Atit & Rocha, 2020). However, the data for this study was collected after the shift to remote instruction for K-12 education due to the COVID-19 pandemic and participating teachers were asked to reflect on their current teaching practice. Prior research suggests that many teachers experience computer anxiety (e.g., Rosen & Weil, 1995; Russel & Bradley, 1997), and remote instruction relies heavily on the use of computers and technological resources to convey content and practices. Some teachers’ computer anxiety may deter them from implementing spatial practices when teaching in online environments, regardless of their spatial skills. Therefore, we hypothesize that there will be no relation between teachers’ spatial skills and their use of spatial pedagogy during remote instruction. We controlled for teachers’ reported gender in all our analyses as gender differences are apparent on many tests of spatial skills (e.g., Miller & Halpern, 2013) and gender ratios vary widely depending on the type of teacher (Atit et al., 2018).

## Methods

This study was approved by the Institutional Review Board at the University of California Riverside (Protocol Number HS-19-201; Title of Study: Examining the Role of Teachers’ Spatial Skills on STEM Teaching and Learning).

### Participants

**Fig. 1:**
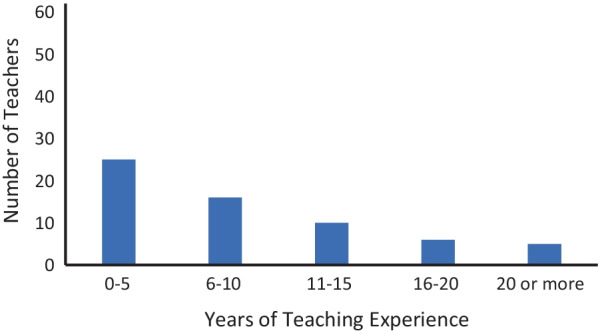
Distribution of teachers across years of teaching experience

Participants of the study included 62 K-12 teachers (male = 15, female = 46, unreported = 1) from across the USA who were teaching remotely. This sample size was deemed adequate as an a priori power analysis estimates that using an alpha of 0.05, a sample of 54 participants would have 80% power to detect a medium effect size (*F* = 0.25) for a linear multiple regression for a fixed model examining for R^2^ increase, with five tested predictors and eight total predictors. Teachers were recruited through social media posts shared by the research team as well as their department’s media and communications teams. The informed consent process was conducted prior to their participation. Participants were compensated with a $25 Target gift card for their participation. The sample consisted of 29 primary teachers, 32 secondary teachers, and one teacher that taught at both educational levels. Twenty-two teachers reported being credentialed to teach single subjects, 36 teachers reported being credentialed to teach multiple subjects, and 4 teachers reported “other” or “none.” A summary of teachers’ reported credential certification type (single or multiple subject) by the educational level (primary or secondary) at which they teach is provided in Table 1. Reported teaching experience ranged from first year teachers to teachers with 20 or more years of experience. The distribution of teachers across years of reported teaching experience is shown in Fig. 1. Teachers’ highest level of educational attainment ranged from bachelor’s degrees to master’s degrees (all teachers reported having earned a bachelor’s degree in arts or sciences, 36 teachers reported earning a master’s degree). A summary of the teachers’ reported educational attainment by the level of K-12 education they teach is provided in Table 2.

**Table 1:** Teachers’ reported type of credentialing by teacher type

Teacher type	Single subject	Multi subject
Primary	4	22
Secondary	18	13
Both	0	1

Teachers who reported “both” were teachers who taught at both the primary and secondary levels concurrently. Four teachers did not report credentialing information or reported “other”

**Table 2:** Teachers’ educational attainment by teacher type

Teacher type	B.A/B.S	MA/MEd	MBA/JD/PhD
Primary	29	15	0
Secondary	32	19	2
Both	1	0	0

“B.A.” denotes Bachelor of Arts, “B.S.” denotes Bachelor of Science, “MA” denotes Master of Arts, “MEd” denotes Master of Education, “MBA” denotes Master of Business Administration, “JD” denotes Juris Doctor, and “PhD” denotes Doctor of Philosophy. Teachers who reported “both” were teachers who taught at both the primary and secondary levels concurrently

### Measures

#### Teaching Background and Demographics Questionnaire

The Teaching Background and Demographics Questionnaire was researcher-created and included five items asking about: gender, teaching credentials acquired (single subject, multiple subject, or other), prior teaching experience, educational attainment, and the educational level they currently teach. In response to the item on the educational level they teach, participants chose from one of the following options: grades K-5 (elementary), grades 6–8 (middle school), or grades 9–12 (high school). Though the grade levels considered primary education versus secondary education may vary by state and school district, here we designated teachers who reported teaching grades K-5 as primary teachers, and teachers who reported teaching grades 6–8 and/or 9–12 as secondary teachers (US Department of Education, 2008). The full Teaching Background and Demographics Questionnaire is provided in “Appendix A.”

#### Mental Rotations Test (MRT)

Participants completed an online version of the Vandenburg and Kuse (1978) Mental Rotations Test (MRT) that was developed by Mitko and Fisher (2020). The Mental Rotations Test is one of the most widely used spatial skills assessments in psychology and education (Lauer et al., 2019; Uttal & Cohen, 2012). In each item of this measure, five-line drawings of 3D forms similar to those used by Shepard and Metzler (1971) were presented. The target form is on the left and four answer choices are on the right. For each of the 24 problems, participants are instructed to select the two choices (out of the four options given) that are identical but rotated versions of the target figure. Keeping with Vandenburg and Kuse’s (1978) scoring methods, participants are only given a point if they correctly identify both matching figures. Thus, scores may range from 0 to 24 points. The test has two parts with 12 problems each. Participants had three minutes to complete each half. Using Cronbach’s *α,* the internal reliability for the measure (*α* = 0.90) was excellent.

#### Teaching Activities Questionnaire—Revised (TAQ-R)

Expanding upon the questionnaire used by Atit and Rocha (2020), the Teaching Activities Questionnaire-Revised (TAQ-R) has 12 items asking about teachers’ use of spatial pedagogical practices (e.g., use of hand gestures to convey complex concepts). For each item, participants were asked to “Please think about your daily teaching experiences. Choose the best response that most closely reflects how often you engage in these activities in your teaching.” Response options included “never,” “once a month,” “once a week,” “three times a week,” or “daily.” These responses were coded from 1 (never) to 5 (daily) for each item and a total sum of the responses for all 12 items was calculated. Using Cronbach’s *α*, the internal reliability for the questionnaire (*α* = 0.82) was very good. The full TAQ-R is provided in “Appendix B.”

#### International Cognitive Ability Resource (ICAR)—Verbal Reasoning & Matrix Reasoning

We employ a verbal reasoning measure and a matrix reasoning measure to assess teachers’ general reasoning ability. Verbal reasoning and matrix reasoning have both been identified as indicators of general intelligence (Harrison et al., 2015; Smedler & Torestad, 1996). The measures used in this study come from a collection of open-source assessments created for public-use by the International Cognitive Ability Resource Project (ICAR; Condon & Revelle, 2014). Neither of these measures had a time limit.

*Verbal reasoning* The verbal reasoning assessment consisted of 16 items made up of a variety of logic, vocabulary, and general knowledge multiple choice items such as, “If the day after tomorrow is two days before Thursday, then what day is it today?” Participants had to choose the correct answer from the following response choices: “(A) Friday (B) Monday (C) Wednesday (D) Saturday (E) Tuesday (F) Sunday.” Participants’ score on this assessment was the sum of the number of correct responses (ICAR, 2014). Using Cronbach’s *α*, the internal reliability for the measure (*α* = 0.40) was extremely poor.

*Matrix reasoning* The matrix reasoning assessment consisted of 11 items that contain stimuli similar to those used in Raven’s Progressive Matrices (ICAR, 2014). Each item shows a 3 × 3 array of geometric shapes where one of the nine shapes is missing. Participants are asked to respond by choosing which of six geometric shapes presented as response choices will best complete the array. Participants’ score on this assessment was the sum of the number of correct responses. Using Cronbach’s *α*, the internal reliability for the measure (*α* = 0.69) was acceptable.

#### Trait Anxiety Inventory

Participants completed the Trait Anxiety Inventory (Spielberger et al., 1970). In this 20-item measure, participants are asked to read each statement and select the response that corresponds to how frequently they experience general feelings of anxiety. A sample item from the measure reads, “I worry too much over something that really doesn’t matter.” The response options are (0) almost never, (1) sometimes, (2) often, (3) almost always. Responses on each item were scored from 0 to 3 and total scores for the measure are calculated by taking the sum of the responses for all items. Scores could range from 0 to 60. Higher scores indicate higher levels of general anxiety. Using Cronbach’s *α*, the internal reliability of the measure (*α* = 0.85) was good.

#### Spatial Anxiety Scale

The Spatial Anxiety Scale consists of 24 items aimed at measuring participants’ anxiety when completing spatial tasks (Lyons et al., 2018). The 24 items can be divided up into three subscales (8 items in each subscale): Mental Manipulations, Imagery, and Navigation. For each item, participants were asked to “mark the response that describes *how much you would be made to feel anxious by it*.” Response options included “not at all,” “a little,” “a fair amount,” “much,” “very much.” The measure was not timed, but participants were asked to work quickly while still thinking about each item. The Mental Manipulation subscale included items where participants were asked to imagine manipulating an object and the spatial relations that define it. The Imagery subscale included items where participants were asked to imagine an object and the spatial relations that define it. Lastly, the Navigation subscale included items where participants were asked to imagine navigating objects (including themselves) and the spatial relations involved in doing so.

Scores on each item ranged from 0 (not at all) to 4 (very much). Subscale scores were calculated by summing the scores for the eight items in each subscale. Furthermore, the score for the total assessment was calculated by summing the scores from all 24 items. Using Cronbach’s α, internal reliability for all subscales combined (*α* = 0.94) was excellent. The internal reliability for each of the subscales were also excellent: *α* = 0.93 for Mental Manipulation, *α* = 0.92 for Imagery, and *α* = 0.91 for Navigation.

### Procedure

Due to the COVID-19 pandemic, all surveys and instruments were administered online via *Qualtrics* software. Prior to completing any measures, participants completed an online informed consent process, in which the consent information was presented, and participants provided or denied consent within the Qualtrics form. After consent was provided, participants then completed the measures in the following order: MRT, TAQ-R, ICAR Verbal Reasoning, ICAR Matrix Reasoning, Trait Anxiety Inventory, Spatial Anxiety Scale, Teaching Background and Demographics Questionnaire. Measures were administered in this order intentionally to reduce the effects of stereotype threat that may result from the collection of demographic information (e.g., Spencer et al., 1999; Steele & Aronson, 1995). Researcher-created materials, the data collected, and the analysis code for this study are available by emailing the corresponding author.

## Results

All analyses were conducted using R version 3.5.3 (R Corp Team, 2019). Before analyzing our data to answer our research questions, preliminary analyses were conducted to identify associations between variables. Table 3 presents the descriptive statistics for the MRT, TAQ-R, ICAR Matrix Reasoning, Trait Anxiety Inventory, and the Spatial Anxiety Scale and each of its subscales. The ICAR Verbal Reasoning measure was excluded from all analyses due its low reliability. To examine the relations between the variables, Pearson’s correlations between all measures, gender, teacher type (primary versus secondary), teachers’ educational level (no graduate degree versus graduate degree), and teachers’ reported credentials (single subject versus multiple subject) were conducted (presented in Table 4). Following Cohen’s (1988) conventions, results indicated that there was a negative moderate correlation between teachers’ gender and teacher type (*r* = − 0.33), as well as their scores on the Imagery subscale (*r* = − 0.26). Teachers’ credentials were positively and moderately correlated with teachers’ gender (*r* = 0.26), as well as Mental Manipulation (*r* = 0.37) and Imagery (*r* = 0.23) subscale scores. Grade level taught was negatively and moderately associated with teachers’ credentialing (*r* = -0.37). Teachers’ Mental Rotation scores showed a moderate negative correlation with their scores on the TAQ-R (*r* = − 0.28), the Spatial Anxiety Scale (*r* = − 0.25), and the Mental Manipulation subscale (*r* = − 0.40), but a moderate positive correlation with their performance on the ICAR Matrix Reasoning (*r* = 0.28). Teachers’ Spatial Anxiety Scale scores were positively and moderately correlated with their scores on the Trait Anxiety Inventory (*r* = 0.26), and positively and strongly correlated with their scores on the Mental Manipulation (*r* = 0.80), Imagery (*r* = 0.74), and Navigation (*r* = 0.85) subscales. These results indicate that teacher type and spatial anxiety for tasks involving imagery did vary by teachers’ gender. Additionally, spatial anxiety for tasks involving mental manipulation of objects and navigation varied by teachers’ reported type of credentialing. Lastly, teachers with stronger spatial skills reported less frequently using spatial pedagogical practices in their teaching in remote learning settings, lower spatial anxiety overall, lower spatial anxiety for tasks involving mental manipulation, and demonstrated higher general reasoning skills.

**Table 3:** Descriptive statistics for all measures

Measure	*M* (SD)	*n*
MRT	7.26 (5.75)	61
TAQ-R	29.42 (8.84)	62
Matrix Reasoning	6.77 (2.61)	62
Trait Anxiety Inventory	19.08 (7.74)	62
Spatial Anxiety Scale	40.97 (17.88)	62
Mental Manipulations	12.68 (7.62)	62
Imagery	14.31 (7.78)	62
Navigation	13.98 (7.07)	62

Mental Manipulations, Imagery, and Navigation are all subscales of the Spatial anxiety Scale

**Table 4:** Correlational data among all examined variables

Variable	1	2	3	4	5	6	7	8	9	10	11
1. Credential											
2. GradDegree	0.12										
3. Gender	**0.26**	− 0.11									
4. Type	− **0.37**	0.08	− **0.33**								
5. MRT	− 0.15	0.13	− 0.15	0.25							
6. TAQ-R	0.13	− 0.03	0.21	− 0.15	− **0.28**						
7. Matrix	− 0.21	0.10	0.03	0.01	**0.28**	− 0.20					
8. TAI	− 0.17	− 0.24	0.11	0.16	− 0.13	0.17	− **0.30**				
9. SAS	0.24	− 0.13	− 0.08	− 0.08	− **0.25**	0.01	− **0.32**	**0.26**			
10. Manipulation	**0.37**	− 0.19	− 0.14	− 0.15	− **0.40**	− 0.06	− **0.46**	0.12	**0.80**		
11. Imagery	− 0.03	0.10	− **0.26**	− 0.03	− 0.07	0.09	− 0.11	0.16	**0.74**	**0.30**	
12. Navigation	**0.23**	− 0.23	− 0.08	− 0.02	− 0.13	− 0.02	− 0.18	0 .35	**0.85**	**0.61**	**0.45**

“Credential” is teachers’ reported type of credentialing. “Type” is the teachers’ reported grade level at which they teach (primary, secondary, or both). “Matrix” denotes the ICAR Matrix Reasoning assessment. “TAI” denotes Trait Anxiety Inventory. “SAS” denotes Spatial Anxiety Scale. “Manipulation,” “Imagery,” and “Navigation” are the three subscales of the Spatial Anxiety Scale. Bivariate correlations significant at the *p* < 0.05 level are indicated in **bold** font

**Table 5:** Results of t tests examining differences between primary versus secondary teachers

Measure	Primary		Secondary		*t*	*d*	*B*_*10*_
	*M*	*SD*	*M*	*SD*			
MRT	5.83	4.91	8.41	6.27	− 1.80	0.46	1.00
TAQ-R	31.03	7.57	27.84	9.83	1.43	0.36	1.63
Matrix	6.72	2.70	6.84	2.60	− 0.18	0.05	3.78
TAI	17.59	8.72	20.50	6.70	− 1.45	0.37	1.59
SAS	42.76	17.10	39.25	18.93	0.76	0.19	3.01
Manipulation	14.10	6.47	11.22	8.45	1.51	0.38	1.48
Imagery	14.62	8.03	14.00	7.78	0.31	0.08	3.68
Navigation	14.03	7.36	14.03	7.01	0.00	0.00	3.83

Primary teachers reported teaching grades K-5 and secondary teachers reported teaching grades 6–8 and/or 9–12. “Matrix” denotes the ICAR Matrix Reasoning assessment. “TAI” denotes Trait Anxiety Inventory. “SAS” denotes Spatial Anxiety Scale. “Manipulation,” “Imagery'', and “Navigation” are the three subscales of the Spatial Anxiety Scale

### Do teachers’ spatial skills and spatial anxiety differ for different types of teachers?

To identify if teachers’ spatial skills and spatial anxiety varied by teacher type, we first conducted independent samples *t* tests examining for differences between primary versus secondary teachers for each measure. Additionally, we calculated the Jeffrey–Zellner–Siow Prior Bayes factors using a scale r of 0.707 for each measure (Rouder et al., 2009). The teacher who indicated “both” with regards to the grade levels they teach was excluded from the analysis. Results from the t tests indicated that there was no difference between primary and secondary teachers on any of the measures administered. However, using Schönbrodt and Wagenmakers (2018) criteria, Bayes factors suggest that there is none to anecdotal evidence in favor of the null hypothesis (i.e., there is not a statistically significant difference between primary and secondary teachers) for the Mental Rotations Test, the TAQ-R, the Trait Anxiety Inventory, and the Mental Manipulation subscale. There is some moderate evidence in favor of the null hypothesis for the Matrix Reasoning, the Spatial Anxiety Scale, the Imagery subscale, and the Navigation subscale (Table 5).

To examine if teachers’ gender, general reasoning skills, and general level of anxiety were occluding any differences in spatial skills and spatial anxiety between the two teacher types (i.e., primary versus secondary teachers), we conducted two regression models in which gender, Matrix Reasoning score, and Trait Anxiety Inventory score were included as covariates, and teacher type was the independent variable. We also controlled for teachers’ reported type of credentials in both models, as well as all subsequent analyses, as correlational results indicated that the Mental Manipulation and Imagery subscale scores varied depending on whether they reported acquiring teaching credentials for a single subject or for multiple subjects. Thus, data from four teachers who did not provide any credentialing information were excluded from all subsequent analyses. Mental Rotation score was the outcome variable for the first model (Model 1) and Spatial Anxiety Scale score was the outcome variable for the second model (Model 2). Results, shown in Table 6, indicate that even after controlling for teachers’ reported type of credentials, gender, general reasoning skills, and general anxiety there are no differences in spatial skills or spatial anxiety between primary and secondary teachers.

**Table 6:** Linear regression models examining if spatial skills or spatial anxiety differ by teacher type

	Model 1			Model 2		
	*b*(SE)	*B*	*p*	*b*(SE)	*B*	*p*
Intercept	3.20(5.83)		0.59	43.40(17.13)		0.01**
Gender	− 1.20(1.88)	− 0.11	0.53	− 5.85(5.54)	− 0.14	0.30
Matrix reasoning	0.62(0.33)	0.25	0.07	− 2.07(0.97)	− 0.30	0.04*
Trait anxiety inventory	− 0.03(0.11)	− 0.11	0.77	0.32(0.34)	0.13	0.35
Teacher type	2.53(1.79)	0.19	0.17	− 3.45(5.27)	− 0.10	0.52
Teacher credential	0.05(1.82)	0.00	0.98	7.38(5.34)	0.20	0.17
*R*^2^	0.15			0.22		

*B* are standardized betas. Numbers in parentheses are standard errors. The outcome variable for Model 1 was Mental Rotation score. The outcome variable for Model 2 was teachers’ Spatial Anxiety Scale score

*p < 0.05, **p < 0.01

### What are the relations between K-12 teachers’ spatial skills and spatial anxiety?

To examine the relations between teachers’ spatial skills and spatial anxiety, four additional regression models were conducted. To ensure that the relations between teachers’ spatial skills and their spatial anxiety is not driven by their reported type of credentials, gender, general reasoning skills, or their base level of general anxiety, we controlled for teachers’ reported credentials, gender, Matrix Reasoning score, and Trait Anxiety Inventory score in all four models. Model 3 examined if teachers’ MRT score was related to their overall SAS score, shown in Table 7. Models 4, 5, and 6 examined if teachers’ MRT score was related to their scores on each of the subscales of the SAS (Mental Manipulations, Imagery, and Navigation respectively), shown in Table 8. The results of these analyses indicate that after controlling for gender, Matrix Reasoning score, and Trait Anxiety Inventory score, MRT score was negatively related to SAS Mental Manipulations subscale score (Model 4), but was not related to SAS score overall (Model 3) or the other two subscales (Models 5 and 6). These results indicate that after accounting for teachers’ reported credentials, gender, general reasoning skills, and general level of anxiety, teachers with higher spatial skills have lower spatial anxiety for tasks involving mental manipulations. However, their spatial skills do not influence their spatial anxiety overall, or for tasks involving imagery or navigation.

**Table 7:** Linear regression model examining the relations between teachers’ spatial skills and spatial anxiety

	Model 3		
	*b*(SE)	*B*	*p*
Intercept	42.43(15.80)		0.01**
MRT	− 0.53(0.40)	− 0.17	0.19
Gender	− 5.82(5.28)	− 0.14	0.28
Matrix reasoning	− 1.74(0.99)	− 0.25	0.09
Trait anxiety inventory	0.30(0.34)	0.12	0.39
Teacher credential	8.14(4.95)	0.22	0.11
*R*^*2*^	0.24		

*B* are standardized betas. Numbers in parentheses are standard errors. The outcome variable for Model 3 was teachers’ Spatial Anxiety Scale score

*p < 0.05, **p < 0.01

**Table 8:** Linear regression models examining the relations between teachers’ spatial skills and the three types of spatial anxiety

	Model 4				Model 5			Model 6		
	*b*(SE)	*B*	*p*		*b*(SE)	*B*	*p*	*b*(SE)	*B*	*p*
Intercept	17.19(6.12)		0.01		18.82(7.09)		0.01	6.43(6.20)		0.01
MRT	− **0.35(0.16)**	− **0.26**	**0.01**		− 0.13(0.18)	− 0.10	0.49	− 0.05(0.16)	− 0.05	0.57
Gender	0.78(2.04)	0.04	0.70		− 4.65(2.37)	− 0.27	0.06	− 1.95(2.07)	− 0.13	0.67
Matrix	− **1.12(0.38)**	− **0.38**	**0.01**		− 0.32(0.44)	− 0.11	0.47	− 0.29(0.39)	− 0.11	0.58
TAI	− 0.05(0.13)	− 0.05		0.68	0.10(0.15)	0.10	0.52	0.25(0.13)	0.27	0.06
Credential	3.87(0.05)	0.24		0.04	0.05(2.22)	0.00	0.98	**4.22(1.95)**	**0.30**	**0.03**
*R*^2^	0.39				0.13			0.12		

*B* are standardized betas. Numbers in parentheses are standard errors. “Matrix” denotes the ICAR Matrix Reasoning assessment. “TAI” denotes the Trait Anxiety Inventory. “Credential” is teachers’ reported type of credentialing. The outcome variables for Models 4, 5, and 6 are teachers’ Mental Manipulation subscale score, Imagery subscale score, and Navigation subscale score in that corresponding order. Significant input variables at the *p* < 0.05 level have been indicated in **bold** font

### How do teachers’ spatial skills and spatial anxiety relate to their use of spatial teaching practices during remote instruction?

To address if teachers’ spatial skills and spatial anxiety were related to their use of spatial pedagogical practices during remote instruction, three additional regression models were conducted. In line with the previous analyses, we controlled for teachers’ reported type of credentials, gender, Matrix Reasoning score, and Trait Anxiety Inventory score, in each model. Model 7 examined if teachers’ MRT score in conjunction with their Spatial Anxiety Scale score predicted their TAQ-R score. Model 8 examined if teachers’ MRT scores in conjunction with their Spatial Anxiety Scale subscale scores (i.e., Mental Manipulation, Imagery, and Navigation) predicted their TAQ-R scores. Results of these analyses, shown in Table 9, revealed that MRT score in conjunction with the Mental Manipulation subscale score negatively predicted TAQ-R score after controlling for teachers’ reported credentials, gender, general reasoning skills, and general anxiety. However, MRT score in conjunction with Spatial Anxiety Scale subscale score was not significantly related to the TAQ-R score.

**Table 9:** Regression analyses examining if teachers’ spatial skills and spatial anxiety predict their use of spatial pedagogical practices during remote instruction

	Model 7			Model 8		
	*b*(SE)	*B*	*p*	*b*(SE)	*B*	*p*
Intercept	30.13(8.79)		< 0.001	32.17(8.65)		< 0.001
MRT	− 0.31(0.21)	− 0.20	0.15	− **0.46(0.21)**	− **0.30**	**0.03**
Gender	3.71(2.78)	0.19	0.19	**6.28(2.77)**	**0.31**	**0.03**
Teacher credential	0.99(2.64)	0.05	0.71	2.38(2.58)	0.13	0.36
Matrix	− 0.55(0.53)	− 0.16	0.30	− 1.04(0.54)	− 0.31	0.06
TAI	0.21(0.18)	0.18	0.24	0.09(0.18)	0.08	0.62
SAS	− 0.08(0.07)	− 0.16	0.29			
Mental manipulation				** − 0.68(0.25)**	− **0.59**	**0.01**
Imagery				0.28(0.17)	0.24	0.11
Navigation				0.13(0.23)	0.10	0.58
*R*^*2*^	0.17			0.29		

*B* are standardized betas. Numbers in parentheses are standard errors. “Matrix” denotes the ICAR Matrix Reasoning assessment. “TAI” denotes Trait Anxiety Inventory. “SAS” denotes the Spatial Anxiety Scale. “Mental Manipulation,” “Imagery,” and “Navigation” are all subscales of the Spatial Anxiety Scale. The outcome variable for Models 7 and 8 is teachers’ score on the Teaching Activities Questionnaire-Revised (TAQ-R). Significant input variables at the *p* < 0.05 level have been indicated in bold font

**Fig. 2:**
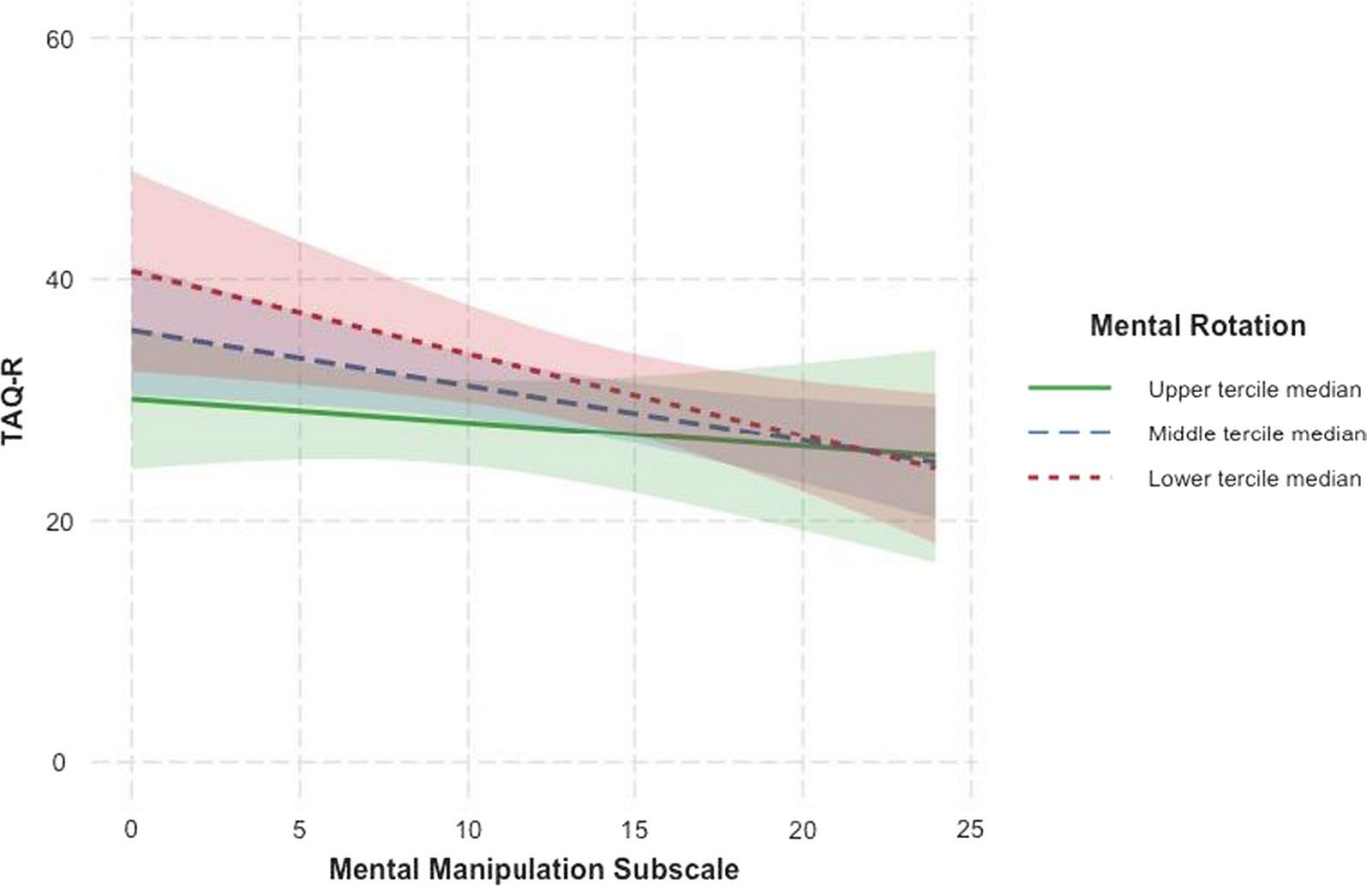
The relations between teachers’ spatial skills, mental manipulation anxiety, and use of spatial pedagogical practices. *Note.* This graph depicts the interaction between teachers’ mental rotation score, Mental Manipulations Anxiety subscale score, and Teaching Activities Questionnaire-Revised score. Since mental rotation score is a continuous variable, the data were split into three equal-sized groups (i.e., terciles) based on teachers’ score on the assessment. The three distinct lines on the graph represent the upper, middle, and lower thirds of the distribution of teachers’ mental rotation score. The corresponding shaded regions signify the 95% confidence interval for each median

To further understand the relations between teachers’ spatial skills and mental manipulation anxiety on their use of spatial teaching practices during remote instruction, we examined if MRT score and Mental Manipulations subscale score interact in Model 9, shown in Table 10. A centered interaction term between MRT score and Mental Manipulations subscale score was included in the model and Imagery and Navigation subscale scores were removed from the model. Analyses revealed a significant effect of Mental Manipulations subscale score, but no effect of MRT score, or no significant interaction between the two factors. These results indicate that teachers with weaker spatial skills and who have lower spatial anxiety for tasks involving mental manipulations report greater use of spatial pedagogical practices in their teaching for online learning environments. However, teachers’ spatial skills and mental manipulation anxiety do not interact to explain their online teaching practices. A graph depicting the relations between teachers’ MRT score, Mental Manipulations subscale score, and TAQ-R score is provided in Fig. 2.

**Table 10:** Regression analyses examining if teachers’ spatial skills and mental manipulation anxiety interact to predict their use of spatial pedagogical practices during remote instruction

	Model 9		
	*b*(SE)	*B*	*p*
Intercept	36.92(8.65)		< 0.001
MRT	− 0.37(0.22)	− 0.23	0.12
Gender	3.05(2.87)	0.15	0.29
Teacher credential	2.15(2.55)	0.12	0.40
Matrix	− 1.01(0.54)	− 0.30	0.07
TAI	0.13(0.17)	0.11	0.46
Mental manipulation	** − 0.45(0.18)**	** − 0.40**	**0.02**
MRT * mental manipulation	0.04(0.03)	0.18	0.22
*R*^*2*^	0.27		

*B* are standardized betas. Numbers in parentheses are standard errors. “Matrix” denotes the ICAR Matrix Reasoning assessment. “TAI” denotes Trait Anxiety Inventory. ““Mental Manipulation” denotes the Mental Manipulation subscale of the Spatial Anxiety Scale. The outcome variable for Model 9 is teachers’ score on the Teaching Activities Questionnaire-Revised (TAQ-R). Significant input variables at the *p* < 0.05 level have been indicated in bold font

## Discussion

Teachers are undoubtedly an integral part of their students’ learning outcomes and experiences (e.g., Aguilar et al., 2021; Ball et al., 2005; den Brok et al., 2004; Perera & John, 2020). With the recent shift to distance learning at the K-12 educational level due to the COVID-19 pandemic (Aguilar et al., 2021; Cardullo et al., 2021), understanding how teachers’ skills and cognition are related to their remote teaching practices is an important task, especially within the domain of spatial skills because of the marked effect that students’ spatial skills have on their success in STEM domains (e.g., Atit et al., 2020; Wai et al., 2009). Starting with their first experiences in school, spatial thinking has been found to be a consistent independent predictor of later STEM achievement (e.g., Simmons et al., 2008) for children in the earliest grades. For example, Jordan et al. (2010) examined number sense and mathematics achievement in 175 students in 1^st^ grade, who were later re-evaluated in 3^rd^ grade. Their results identified that spatial skills were a significant predictor for student mathematics achievement across grade levels. Further, classroom interventions introducing spatial strategies suggest that children can improve their mental rotation and other spatial skills when practice is introduced at even the earliest of school ages (ages 4 ½–5: Ehrlich et al., 2006; ages 6–8: Cheng & Mix, 2014; ages 5–10: Gilligan et al., 2017). These findings indicate that primary school and secondary school teachers have an important role in setting the foundation of these abilities if they choose to include them as part of their pedagogical practices. Thus, understanding the factors that help explain teachers' decisions to incorporate practices that leverage and bolster students’ spatial skills from those practices that do not, and if these factors differ for primary and secondary teachers and how to address them, becomes an essential requirement for fostering the delivery of more supportive STEM learning environments for both in-person and remote learning settings.

Therefore, building on prior research examining the teachers’ role in students’ spatial learning (e.g., Atit & Rocha, 2020; Gunderson et al., 2013; Otumfuor & Carr, 2017), we investigated the relations between K-12 teachers’ spatial skills, their spatial anxiety, and the factors influencing their use of spatial pedagogical practices during remote instruction. Our findings reveal that spatial skills and spatial anxiety may not vary between primary and secondary teachers. However, teachers with higher spatial skills showed lower spatial anxiety for tasks involving mental manipulations. Additionally, teachers’ spatial skills and their anxiety for mental manipulation tasks predicted their reported use of spatial pedagogical practices during remote instruction. Specifically, teachers with weaker spatial skills and lower mental manipulation anxiety reported more frequently using spatial teaching practices.

Contrary to the findings of prior research on the differences in spatial skills between teacher types (Atit et al., 2018), our results suggest that spatial skills may not vary between primary and secondary teachers. Atit et al. (2018) examined differences in spatial skills in high schoolers who later became preschool/primary, secondary STEM, or secondary non-STEM teachers. Preschool/primary teachers had significantly weaker spatial skills than secondary STEM teachers but did not differ from secondary non-STEM teachers. The differences between Atit et al.’ (2018) findings and the study presented here could be attributed to the fact that the data examined by Atit et al. (2018) was collected from high school students in the 1960s who were future teachers. Whereas in this study, the data examined were collected from current practicing teachers. STEM content at the primary level lends itself to being taught using spatial tools, such as manipulatives and diagrams. For example, the water cycle and other complex systems are often taught in the classroom by engaging with visual diagrams (Lee et al., 2019; Smith & Samarakoon, 2014). Additionally, the incorporation of the multi-state K-12 Next Generation Science Standards (NGSS; NGSS Lead States, 2013) since the 1960s sampling has encouraged this usage. The NGSS are a revision of the list of skills and knowledge students should be able to perform across the four main science subject areas (Physical Science, Life Science, Earth and Space Science, and Engineering Design). While spatial learning has not been incorporated as a formal skill to be taught explicitly by teachers, spatial reasoning concepts such as developing and using models and diagrams appear throughout the disciplinary core ideas forming the framework of the NGSS standards. For example, primary teachers may be practicing using more geospatial models than previously as they address the disciplinary core idea of “the sun is a star that appears larger and brighter than other stars because it is closer” that underlies the fifth grade NGSS standard 5-ESS-1 stating “support an argument that differences in the apparent brightness of the sun compared to other stars is due to their relative distances from Earth.” Research on spatial skills indicates that spatial skills are malleable and can be improved through training and practice (Uttal et al., 2013). Thus, experience teaching primary STEM content using spatial tools may have resulted in diminishing differences between primary and secondary teachers’ spatial skills in this study. Due to our small sample size, we were unable to differentiate between secondary STEM and secondary non-STEM teachers. Thus, it is unknown if current primary teachers’ spatial skills differ from those of teachers who teach STEM content or non-STEM content at the secondary level. Future research should examine if differences between primary and secondary teachers’ spatial skills are apparent when considering their content specialties.

To our knowledge, no prior studies have compared levels of spatial anxiety between teacher types. In our study, we examined if spatial anxiety differed between primary and secondary teachers and found no significant differences between the two groups. These findings are informative as they build on research by Gagnier et al. (2021) who found that primary teachers experience low anxiety when solving spatial problems and believe that their spatial skills can improve with practice. However, when asked about their confidence in being able to cultivate students’ spatial skills during science instruction, teachers reported lower self-efficacy than their efficacy for teaching science or teaching in general (Gagnier et al., 2021). Taken together, these findings indicate that spatial anxiety for tasks teachers carry out for themselves versus teaching spatial skills in the classroom may be distinct factors. Recent research by Burte et al. (2020) indicates that primary teachers with lower spatial anxiety also exhibit lower anxiety about teaching math, and high math anxiety in primary teachers has been found to hinder math achievement in their students, specifically their girl students (Beilock et al., 2010). Identifying if teachers’ spatial anxiety and anxiety for implementing spatial pedagogy are separable may have broad implications for K-12 students’ STEM learning and thus, is a critical question for future research.

Additionally, one thing to note is our findings regarding the lack of differences between teacher types and their spatial skills, spatial anxiety, and spatial teaching practices warrant further investigation. Results of inferential analyses using significance testing revealed no differences between teacher types on any of the administered measures. However, examination of Bayes factors revealed that there was *none* to *anecdotal* evidence in support of the null hypothesis (i.e., there is no statistically significant difference between primary and secondary teachers), for teachers’ spatial skills, use of spatial pedagogical practices, and anxiety for spatial tasks involving mental manipulation. The large variability in teachers’ scores on these measures, evidenced by the large standard deviations, may have masked the differences between the two groups. Larger sample sizes would increase the statistical power, allowing for a better detection of differences between groups if they do exist (Case & Ambrosius, 2007). Future research examining differences in teacher characteristics and practices between teacher types should aim to recruit larger samples of primary and secondary teachers to verify the findings presented here.

Consistent with prior findings on the relations between teachers’ spatial anxiety and their spatial skills (Atit & Rocha, 2020), results from this study confirmed that teachers’ anxiety for mental manipulation tasks is negatively related to their mental rotation skills. Moreover, the findings reported here confirmed that the negative relation between mental rotation skills and teachers’ mental manipulation anxiety remained even after accounting for teachers’ reported teaching credentials, gender, general reasoning skills, and their general anxiety. However, consistent with prior findings (Atit & Rocha, 2020), no relation between teachers’ mental rotation skills and their anxiety for imagery tasks or navigation tasks was found. It has been well established that spatial skills consist of more than one type of skill (Linn & Petersen, 1985; McGee, 1979), and that teachers’ spatial skills and spatial anxiety are related to their practice as well as their students’ spatial learning (e.g., Atit & Rocha, 2020; Gunderson et al., 2013; Otumfuor & Carr, 2017). In this study, only teachers’ mental rotation skills were examined. Thus, it is unknown if and how teachers’ navigation skills and their spatial imagery skills relate to their spatial anxiety for navigation tasks and imagery tasks. Future research should administer a broad range of spatial skills measures to gain a better understanding of the relations between teachers’ spatial skills and their spatial anxiety.

While the relations between teachers’ spatial skills and spatial anxiety reported here were consistent with earlier studies (Atit & Rocha, 2020), the relations between teachers’ spatial skills, spatial anxiety, and their spatial pedagogical practices contradict earlier findings (Atit & Rocha, 2020). The study reported here found that teachers’ mental rotation skills and their anxiety for mental manipulation tasks were negatively related to their reported use of spatial tools and practices during remote instruction. Specifically, teachers with weaker mental rotation skills and lower mental manipulation anxiety reported more frequently using spatial teaching practices. However, in the study by Atit and Rocha (2020), only teachers’ mental rotation skills predicted their use of spatial teaching tools, and the relation was in the positive direction. Teachers with stronger mental rotation skills reported more frequently using spatial tools in their teaching.

The differences in findings between these studies could be attributed to the following factors. First, in this study, we asked teachers to reflect upon their use of spatial teaching practices during a period of exclusively remote instruction. In the study by Atit and Rocha (2020), teachers reported about their use of spatial teaching practices when in-person classroom instruction was the norm. Perhaps the constraints associated with providing instruction in remote learning settings led teachers with weaker spatial skills and lower mental manipulation anxiety to implement more spatial teaching practices to deliver the necessary content. As discussed earlier, much of the K-12 curriculum (e.g., NGSS) in the USA requires teachers to convey spatially-demanding content and engage students in spatially-demanding tasks. Remote instruction requires teachers to convey much of this content using computer software, apps, and video conferencing platforms. Research suggests that there is a link between one’s spatial skills and their proficiency for spatial language (e.g., Gilligan-Lee et al., 2021; Pruden et al., 2011). Perhaps teachers with weaker spatial skills, but who are not as anxious about engaging in spatial tasks, were more likely to use spatial teaching strategies, such as simulations, computer generated motion, and diagrams, to convey this spatial content due to it being generated by outside sources rather than relying on their own spatial language skills to convey spatial meaning. On the other hand, the broad range of pedagogical strategies available to teachers during in-person classroom instruction may be hindering the selection of spatial teaching practices in teachers with weaker spatial skills even with low spatial anxiety because a wider range of non-spatial strategy options are available to select from. More research is needed to fully understand how the constraints and computer-based resources associated with remote instruction in conjunction with teachers’ characteristics explain teachers’ choice of pedagogical strategies.

Second, in this study, we administered a revised and expanded version of the Teaching Activities Questionnaire. In the revised version, we included seven additional items, and all of the items were rewritten to specifically probe teacher use of spatial practices as well as encouraging students’ engagement with spatial practices as part of their teaching. In the seven new items, we also examined teachers’ use of additional spatial tools, such as analogy (Gentner, 1983) and dynamic visuals (e.g., simulations; Stieff & Wilensky, 2003), which were not examined in the original version. Additionally, Atit and Rocha (2020) did not control for teachers’ level of general anxiety or their general reasoning skills, which were both examined and accounted for in the study reported here. Future research should examine if and how differences in the measures administered contributed to the differences in the findings between studies.

One limitation of this study is that we implemented a self-report measure to assess how often teachers used spatial pedagogical practices during remote instruction. Research on teachers’ accuracy for self-reporting about instructional practices is mixed (Koziol & Burns, 1986; Mayer, 1999), indicating that asking teachers to reflect and report on their use of spatial practices in their teaching may not be the most accurate reflection of the activities taking place. Future research should collect converging evidence using observational methodology to provide a more objective and potentially accurate measure of teachers’ use of spatial pedagogical practices during online teaching.

Another limitation of the study is that while we asked teachers about the type of credentialing they acquired to become a K-12 teacher, the response they provided may not fully reflect the kind of professional training they received. In most states, single subject credentialing programs prepare prospective teachers to teach middle school or high school students a specific secondary subject, while multiple subject credentialing programs prepare prospective teachers to teach students in elementary or primary schools (e.g., Commission on Teacher Credentialing, 2016). Our data show that thirteen secondary teachers reported multiple subject credentialing instead of single subject credentialing. We speculate that they may have chosen that they have multiple subject credentialing for a few reasons. First, we did not explicitly define what we meant by teaching credentials which may have led to some confusion as credentialing requirements vary across states (TEACH, 2021). Second, those teachers may have earned credentials for more than one subject area (e.g., history and English or chemistry and physics) and interpreted *multiple subject* credentialing to mean that they are authorized to teach more than one domain. Third, they may have originally acquired a multiple subject credential and then became authorized afterwards with a second single subject credential over the span of their teaching career and chose to report their original credential when asked in our study. Thus, we are unable to make any conclusions about teachers’ acquired credentials and their use of spatial pedagogical practices during remote teaching. A critical area for future research is the role of prospective teacher training programs on teachers’ use of spatial practices during instruction.

## Conclusions

In conclusion, this study highlights the need to further examine the role of all K-12 teachers’ spatial skills and feelings regarding spatial thinking when considering how to improve students’ spatial and STEM learning. Prior research on the teachers’ role in the development of students’ spatial skills has focused on primary teachers (e.g., Burte et al., 2020; Gagnier et al., 2021; Gunderson et al., 2013) or secondary teachers (e.g., Otumfuor & Carr, 2017; Power & Sorby, 2020) separately and often only within a given content area (e.g., mathematics). Few studies have examined the role of teacher characteristics on the use of spatial pedagogical practices across both teacher types (e.g., Atit & Rocha, 2020). Our study revealed that spatial skills, spatial anxiety, or reported use of spatial pedagogy during remote instruction may not differ between teachers engaged in teaching different educational levels (primary versus secondary). Moreover, our results show that for instances of distance learning, teachers’ reported use of spatial pedagogical practices varies depending on their spatial skills and spatial anxiety. These findings underline that supporting the development of students’ spatial learning in physical and virtual classrooms requires efforts which consider relevant teacher characteristics (i.e., their spatial skills and spatial anxiety) and includes teachers from across the K-12 educational levels.

## Data Availability

All data generated and analyzed during the current study are available from the corresponding author on reasonable request.

## Appendix A

### Teaching background and demographics questionnaire

Please fill out the following questions to the best of your knowledge. If you do not wish to provide an answer to any of the questions, that is okay—feel free to leave it blank.

1. Please select the gender you identify with.
  - Male
  - Female
  - Non-binary
  - None of the above
2. How long have you been teaching for?
  - 0–5 years
  - 6–10 years
  - 11–15 years
  - 16–20 years
  - 20 years or more
3. Please select all of the degree(s) you hold.
  - Bachelor of Arts
  - Bachelor of Science
  - Master of Arts (M.A.)
  - Master of Science (M.S.)
  - Master of Business Administration (MBA)
  - Master of Education
  - Doctor of Education (EdD)
  - Juris Doctor (J.D.)
  - Doctor of Philosophy (PhD)
4. Please select the option(s) that best describe your teaching credentials. If you selected single subject, please select the subject(s) of focus.
  - Multiple subject
  - Single subject
    (i) English
    (ii) Mathematics
    (iii) Social Science
    (iv) Sciences
    (v) World Languages
    (vi) Other
5. What grade level of K-12 schooling do you teach?
  - K-5 (elementary)
  - 6–8 (middle school)
  - 9–12 (high school)

## Appendix B

### Teaching activities questionnaire-revised

Please think about your daily teaching experiences. Choose the best response that most closely reflects how often you engage in these activities in your teaching. Your response options for each item are the following: never, once a month, once a week, three times a week, every day.

1. How often do you use graphs or visual diagrams in your teaching?
2. How often do you ask your students to interpret graphs or visual diagrams?
3. How often do you ask your students to create graphs of visual diagrams?
4. How often do you draw or sketch when explaining complex concepts in your teaching?
5. How often do you encourage your students to draw or sketch to find a solution to a problem?
6. How often do you use manipulatives (e.g., virtual models, physical models, blocks, puzzles, *k’nex*, etc.) when explaining complex concepts in your teaching?
7. How often do you encourage your students to interact with manipulatives (e.g., virtual models, physical models, blocks, puzzles, *k’nex*, etc.) to reason about a concept or to find a solution to a problem?
8. How often do you purposefully use gestures (hand movements) to help convey complex concepts in your teaching?
9. How often do you encourage your students to purposely use gestures (hand movements) when trying to understand complex concepts or when solving a problem?
10. How often do you employ analogies when teaching complex concepts (e.g., water flowing through a pipe would be used to explain blood flowing in a blood vessel)?
11. How often do you employ dynamic visuals when teaching about dynamic concepts and processes (e.g., spinning a globe or using a simulation to show the earth’s rotation about its axis)?
12. How often do you ask students to visualize or imagine the movement of objects when teaching dynamic concepts and processes (e.g., imagine the orbit of the earth around the sun)?

## Abbreviations

STEM: Science, technology, engineering, and mathematics
K-12: Kindergarten to 12th grade
3D: Three-dimensional
2D: Two-dimensional
MRT: Mental Rotations Test
ICAR: International Cognitive Ability Resource
B.A.: Bachelor of Arts
B.S.: Bachelor of Science
M.A.: Master of Arts
MEd: Master of Education
MBA: Master of Business Administration
JD: Juris Doctor
PhD: Doctor of Philosophy
TAQ-R: Teaching Activities Questionnaire-Revised
TAI: Trait Anxiety Inventory
SAS: Spatial Anxiety Scale
NGSS: Next Generation Science Standards
